# Direct Membrane Filtration of Municipal Wastewater: Studying the Most Suitable Conditions for Minimizing Fouling Rate in Commercial Porous Membranes at Demonstration Scale

**DOI:** 10.3390/membranes13010099

**Published:** 2023-01-12

**Authors:** Pau Sanchis-Perucho, Daniel Aguado, José Ferrer, Aurora Seco, Ángel Robles

**Affiliations:** 1 CALAGUA–Unidad Mixta UV-UPV, Departament d’Enginyeria Química, Universitat de València, 46100 Burjassot, Spain; 2 CALAGUA–Unidad Mixta UV-UPV, Institut Universitari d’Investigació d’Enginyeria de l’Aigua i Medi Ambient–IIAMA, Universitat Politècnica de Valencia, 46022 Valencia, Spain

**Keywords:** membrane fouling control, microfiltration, primary settled wastewater, raw sewage, ultrafiltration

## Abstract

This study aimed to evaluate the feasibility of applying a commercial porous membrane to direct filtration of municipal wastewater. The effects of membrane pore size (MF and UF), treated influent (raw wastewater and the primary settler effluent of a municipal wastewater treatment plant) and operating solids concentration (about 1 and 2.6 g L^−1^) were evaluated on a demonstration plant. Filtration periods of 2–8 h were achieved when using the MF membrane, while these increased to 34–69 days with the UF membrane. This wide difference was due to severe fouling when operating the MF membrane, which was dramatically reduced by the UF membrane. Use of raw wastewater and higher solids concentration showed a significant benefit in the filtration performance when using the UF module. The physical fouling control strategies tested (air sparging and backwashing) proved to be ineffective in controlling UF membrane fouling, although these strategies had a significant impact on MF membrane fouling, extending the operating period from some hours to 5–6 days. The fouling evaluation showed that a cake layer seemed to be the predominant reversible fouling mechanism during each independent filtration cycle. However, as continuous filtration advanced, a large accumulation of irreversible fouling appeared, which could have been related to intermediate/complete pore blocking in the case of the MF membrane, while it could have been produced by standard pore blocking in the case of the UF membrane. Organic matter represented more than 70% of this irreversible fouling in all the experimental conditions evaluated.

## 1. Introduction

Global water stress and climate change have been identified as two of the most important worldwide problems to be addressed in the next few decades [1,2]. Continuous use of non-renewable resources in the current economic models demonstrates unsustainability in the long-term, boosting the need to find new water, energy and nutrient sources to cope with the increasing demands for these essential resources. Due to this, new economic models based on the circular economy (CE) need to be adopted [3] while developing more energy-efficient and greener technologies to achieve self-sufficient sustainable systems. In the sewage treatment sector, the municipal wastewater (MWW) paradigm has changed dramatically in recent years and is starting to be considered not as a waste but as a source of vital resources, including reclaimed water, energy and nutrients [4]. New membrane-based technologies and alternative treatment schemes are being proposed to transform the former concept of wastewater treatment plants (WWTP) into new resource recovery facilities, such as membrane photobioreactors [5,6], anaerobic membrane bioreactors [7] (also including alternative treatment platforms and configurations [8]) and other advanced systems for water and nutrient recovery [9,10,11]. Moreover, produced water quality is being improved by emerging MWW tertiary treatment for effectively removing antibiotics and other emerging contaminants, such as photodriven advanced oxidation and photocatalysis [12,13,14]. Among the new MWW treatment alternatives, direct filtration of MWW, commonly referenced by the scientific community as direct membrane filtration (DMF), has recently gained increasing interest [15]. This alternative consists of using membrane technology to capture and concentrate the organic particulate fraction of the influent MWW to reduce/avoid the energy demands of secondary treatments (reduction in oxygen requirements of aerobic microorganism included in classic sewage treatment scheme) while boosting the amount of energy that can be recovered from anaerobic digestion (AD) by increasing the organic matter intake. Numerous studies have been carried out to date aimed at determining the feasibility of applying the DMF concept to MWWs [16]. Unfortunately, severe fouling is usually reported when directly filtering sewage by porous membranes [17,18], so implementation of effective strategies for minimizing membrane fouling is an imperative matter to enable application of the DMF alternative [19].

**Figure 1:**
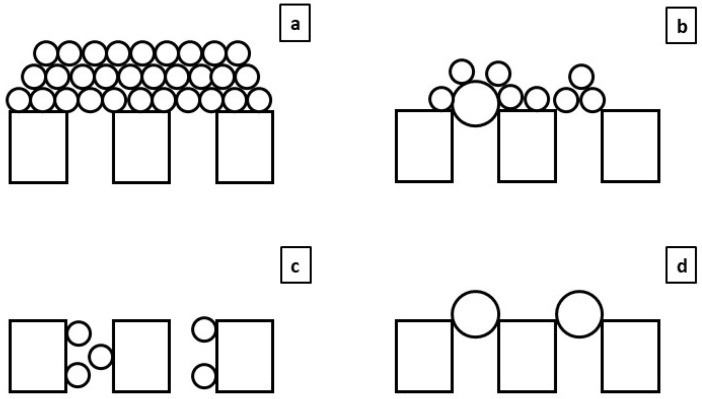
Fouling mechanism graphical scheme: (**a**) cake layer, (**b**) intermediate pore blocking, (**c**) standard pore blocking and (**d**) complete pore blocking.

Fouling in porous membranes can generally be classified into two categories: cake layer and pore blocking [20]. The former describes accumulation of particulate material on the membrane surface as filtration advances and is usually associated with reversible fouling in proportion to the applied flux. Conversely, the latter describes partial/complete obstruction of the membrane pores by deposition of colloidal particles or sticky gel formations, which rapidly hinders membrane permeability. This type of fouling can be either reversible or irreversible depending on the difficulty of removing the adhering substances. Pore blocking can be divided into different sub-categories depending on the nature of the useful membrane area affected, differentiating between intermediate pore blocking (some particles can block the membrane pores or be accumulated onto other particles forming a pseudo cake layer), standard pore blocking (a reduction in membrane pore size due to accumulation of materials inside the membrane pores) and complete pore blocking (the particles accumulate only on the membrane surface, completely blocking the membrane pores). Figure 1 shows a graphic scheme of the different types of fouling.

In the case of the DMF of MWW, the main source of the reported severe fouling has not yet been clearly described, so further studies are required to minimize fouling during filtration. First, the appropriate operating conditions need to be determined for passively minimizing fouling when directly filtering MWW. In this regard, several authors have studied different membrane technologies to apply the DMF alternative, generally using MF and UF membranes due to their larger pore sizes [19]. Nonetheless, the different operating conditions used in the different studies (i.e., filtering flux, filtration–relaxation periods, influent characteristics, physical fouling control strategies, etc.) hinder attainment of proper conclusions such that further studies are needed to compare the real potential of each membrane technology. Use of more refined influent for applying the DMF, such as primary settler effluent (PSE), has also been suggested for reducing membrane fouling [17,21]. This strategy could be an interesting approach since it takes advantage of the existing facilities and a large amount of the influent MWW particulate material would be recovered in a previous step, reducing the amount of solids in contact with the membrane and presumably reducing the treated MWW fouling potential. Additionally, the sludge recovered during filtration can be mixed with sludge from the primary settler for direct use via AD and would not affect the energy recovery potential. This alternative scheme would provide important potential benefits and needs to be studied in depth to properly compare the overall energy and resource recovery that can be obtained regarding the influent used (i.e., raw and PSE MWW).

Diverse physical and chemical fouling control strategies have been proposed by numerous authors for minimizing fouling growth, thereby avoiding short-term chemical cleaning [16,17]. Nonetheless, some of the proposed on-line methods, although effective, are energy-intensive applications that would dramatically reduce the resource recovery potential of the DMF alternative. Different fouling control strategies, therefore, need to be tested under diverse operating conditions to determine the best methods of minimizing fouling while reducing the filtration energy input. Other crucial operating conditions, such as effective operating solids concentration, need to be considered and evaluated to boost resource recovery and minimize energy demands. In this regard, low operating solids concentrations would presumably involve lower filtration energy inputs since less filtering resistance is expected. However, the recovered sludge would require post-treatment for valorisation, increasing the resources required for feeding the AD process (e.g., pumping demands, space requirements, etc.), while high operating concentrations may require higher energy filtration requirements but could reduce or even eliminate any additional steps to recover the sludge. Thus, the overall process should be considered to properly determine the most appropriate treatment scheme.

Several studies have explored the best/optimal operating conditions to minimize fouling when filtering MWW using porous membranes. However, most of them have been conducted at lab-/bench-scale, so further studies are needed in order to assess the feasibility of the technology on a semi-industrial scale. Moreover, the effect of the main operating conditions on membrane fouling cannot be directly extrapolated from lab- to industrial-scale because it depends heavily on membrane configuration and size/length. For instance, the effect and efficiency of air sparging upon membrane fouling depends considerably on the fibre length and hydrodynamic conditions in the membrane tank. On the other hand, a proper comparison between MF and UF technology using comparable influent and operating conditions (operating flux, fouling control strategies, etc.) has not been performed yet.

In this work, a demonstration prototype was operated to determine adequate operating conditions to minimize fouling when filtering MWW using porous membranes. The influences of membrane pore size (MF and UF membranes), treated influent (raw and PSE MWW) and operating total suspended solids (TSS) (about 1 and 2.6 g L^−1^) were evaluated. Additionally, the efficiency of two physical fouling control strategies (air scouring and permeate backwashing) was assessed for each operating scenario tested. On the other hand, chemical cleaning analysis and theoretical filtration modelling exercises were conducted to determine/elucidate the origin (inorganic, organic) and mechanism (cake layer, intermediate/complete/standard pore blocking) of fouling occurring in each of the tested alternatives, thus providing data for developing future fouling control strategies aimed to optimize systems of this type. This plant was fed with wastewater coming from the “Conca del Carraixet” WWTP (Valencia, Spain). The design characteristics and scale of this plant are considered adequate to generate good performance data for scaling up the evaluated technology to full-scale UWW treatment since it incorporates commercial full-scale hollow-fibre membrane modules and equipment.

## 2. Materials and Methods

### 2.1. DMF Plant

**Figure 2:**
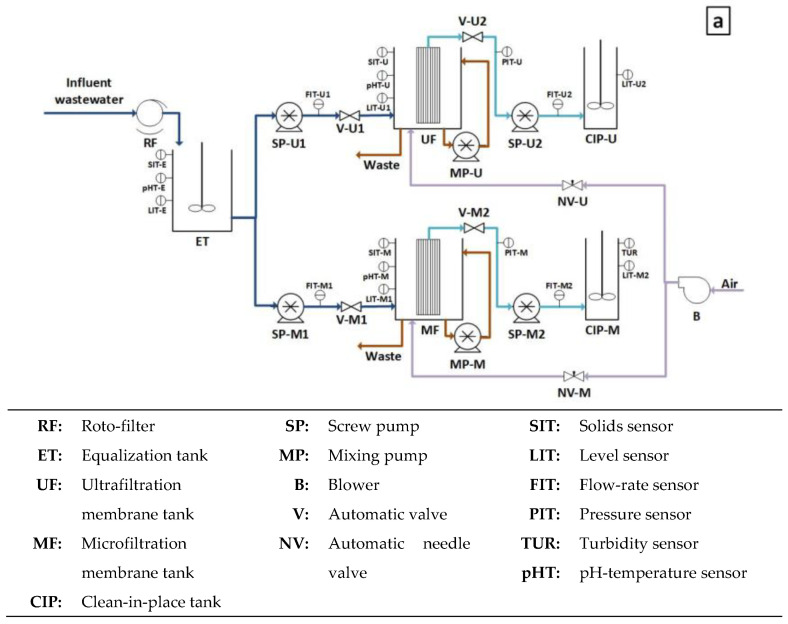

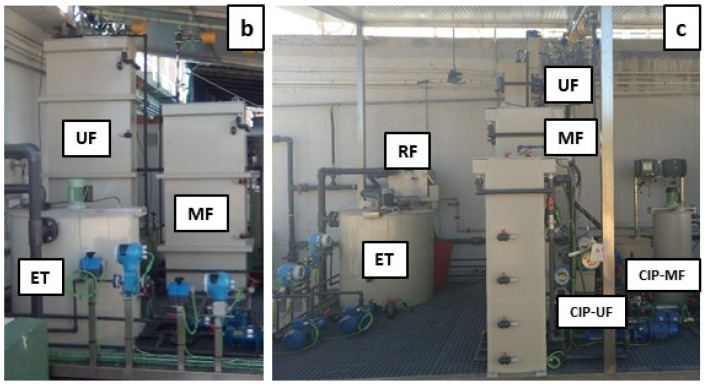
DMF demo-scale plant: (**a**) diagram scheme, (**b**) front view and (**c**) side view.

Figure 2a shows a flow diagram of the DMF plant used in this study, which consisted of two independent membrane tanks fitted with two different industrial membrane systems: MF (TERAPORETM 5000, 0.4-µm pore size, filtration area 18 m^2^, initial tap water permeability of 455 L per bar m^2^ h) and UF (PULSION^®^ Koch Membrane Systems, 0.03-µm pore size, filtration area 43.5 m^2^, initial tap water permeability of 294 L per bar m^2^ h), each equipped with a screw pump (PCM M series, EcoMoineau™) for vacuum filtration. The membrane tanks had a clean-in-place (CIP) tank (100 and 400 L for the MF and UF CIP tanks, respectively) to store the generated permeate and allow membrane backwashing during continuous operation. Air scouring was used to mitigate fouling during filtration using a blower (G-BH7, Elmo Rietschle) to inject air from below each membrane tank. Two additional mixing pumps (PCM M series, EcoMoineau™) continuously recirculated the content of each module to ensure complete mixing of the membrane tanks and avoid solids stratification.

The plant was continuously fed by MWW from the “Conca del Carraixet” full-scale WWTP (Alboraya, Spain). Two different influents were used during this study: (1) raw MWW (after the typical screening and sieving, desanding and degreasing pre-treatment) and (2) PSE from the WWTP. Table 1 shows a characterization of both influents. Regardless of the influent used, a 0.5 mm screen-size roto-filter was installed as pre-treatment to protect the membrane modules, collecting the pre-treated influent in an equalization tank (745 L) to feed each membrane tank with the same MWW. Two additional screw pumps (PCM M Series, EcoMoineau™) were used in each membrane tank to continuously replenish the permeated volume with fresh MWW according to filtration requirements. Figure 2b,c shows views of the system.

### 2.2. Instrumentation, Automation and Control

The DMF plant was designed with a high level of automation. Numerous on-line sensors and automatic equipment were installed to allow full control of all its functions (see Figure 2a). The on-line sensors were: three pH-temperature sensors (InPro3100/120/PT100, Endress + Hauser) in the ET, MF and UF tanks; five level sensors (Cerabar PMP11, Endress + Hauser), one for each tank: ET, MF, UF, CIP-MF and CIP-UF; two liquid pressure sensors (IP65, Druck) to control the TMP in each membrane tank during filtration; four liquid flow meters (Picomag, Endress + Hauser), each one associated with an individual screw pump; two air flow meters, one for each membrane module: MF and UF; three solids sensors (LXV424.99.00100, Hach) to control the solids concentration in the ET, MF and UF tanks and one turbidity sensor (LXV424.99.00100, Hach) to control the turbidity level in the CIP-MF tank. On the other hand, the plat was equipped with the following actuators: five frequency converters (SINAMICS G120C, Siemens), four to control the liquid flowrate of each liquid pump and one to control the air flowrate supplied by the blower; two automatic needle valves to accurately control the air flow-rate distributed to each membrane tank and four on–off control valves to avoid liquid fluxes during relaxation stages. All these instruments were connected to a programmable logic controller (PLC) for proper multi-parametric control and data acquisition. The PLC was also connected via Ethernet network to a PC from which a SCADA system centralized all the information collected by the instruments and facilitated their supervision and control.

Due to the significant number of sensors and actuators installed, the control script was based on multiple control loops, which consisted of classic PID and on–off controllers designed to act on the main operational variables (e.g., liquid and air flow rates, TMP control, level measures in each tank, etc.) to reach the established set-points.

### 2.3. Plant Operation and Experimental Plan

The system was operated continuously, performing consecutive filtration:relaxation cycles. The filtration cycle duration was set to 300 s and relaxation duration was set to 60 s, operating at a filtration–relaxation ratio of 5:1. The operation flux was set to 10 LMH (L per m^2^ and h) for both membranes during all the experiments performed. Air sparging and permeate backwashing were used as fouling control strategies during filtration for both membrane technologies studied. Air sparging intensity was set at a low specific air demand (SAD) of 0.1 m^3^ m^−2^ h^−1^ for minimizing the filtration energy demands, while backwashing was also set to a relatively low periodicity (2 min of backwashing every 10 cycles of filtration:relaxation, therefore operating at a filtration:relaxation:backwashing ratio of 50:10:2) to maintain low physical cleaning downtimes of total operating time. Both air sparging and backwashing were kept constant during all the experiments and were only increased at the end of every experimental filtration period to test their effectiveness for reducing fouling. Each membrane module was operated at a constant TSS concentration depending on the experiment performed. Since the TSS in the waste increases during filtration due to retention of particulate material, part of the sludge produced was continually purged from the membrane tanks, adjusting this purge as necessary to operate at the required TSS concentration.

Different operating/design conditions were tested to determine the most suitable ones for minimizing fouling when filtering MWW: membrane pore size (MF and UF membranes), treated influent (raw and PSE MWW) and operating TSS concentration (about 1 and 2.6 g L^−1^). Table 2 summarizes all the experiments performed. Regarding the different TSS concentrations, the membrane tanks were operated without waste production until the desired concentration was reached (from some hours to a maximum of 1.2 days depending on the concentration desired), later performing continuous waste purge to maintain a constant TSS concentration during filtration. However, due to the severe fouling growth rate when operating the MF membrane, this module was initially fed with the UF membrane waste when necessary (2.6 g L^−1^ TSS concentration experiments) to achieve the required TSS concentration in this membrane tank without losing a significant fraction of its permeability during the concentrating time.

### 2.4. Analytical Methods and Calculations

Each treated influent was sampled once a week to characterize the main pollutants. The UF membrane concentrated sludge and generated permeate were sampled twice a week, while sampling was performed every day in the MF membrane due to severe fouling. Solids, total and soluble chemical oxygen demand (COD and SCOD), total nitrogen (TN) and total phosphorus (TP) were determined according to standard methods [22]. 0.45-mm pore size glass fibre membrane filters (Millipore, Merck) were used to produce the soluble fraction of collected samples.

To assess the filterability of the concentrated sludge in the UF membrane, soluble microbial products (SMP) concentration, viscosity and time to filter (TTF) were determined twice a week. The effective SMP concentration was attributed to protein and carbohydrate concentrations only. A commercial total protein kit (Micro Lowry Peterson’s Modification, St. Louis, MO, USA, Sigma-Aldrich) was used to determine the protein content, while carbohydrates were determined by the Dubois method [23]. The treated sludge viscosity was determined by a Cannon-Fenske viscometer (Series 50, COMECTA^®^), while the TTF was performed according to standard methods [22]. The particle size distribution of the treated influents and TSS concentrations tested in the UF membrane were determined by a laser granularity distribution analyser (Malvern Mastersizer 2000; detector range of 0.01 to 1000 µm).

Different theoretical equations were used to study the predominant fouling mechanism involving direct filtration of MWW. As proposed by Fujioka and Nghiem [20] and Ho and Zydney [24], the general fouling mechanism (i.e., cake layer, intermediate pore blocking, standard pore blocking and complete pore blocking) can be mathematically modelled by the following expression:

(1) $$\frac{d\;TMP_{t}}{d\;t}=K{\left(TMP_{t}\right)}^{n}$$

where *t* represents the filtration time, *TMP_t_* is the transmembrane pressure at each instant, *K* is a fouling law constant and *n* represents the fouling index, which can take different values depending on the dominant fouling mechanism (*n* = 0 for cake layer, *n* = 1 for intermediate blocking, *n* = 1.5 for standard blocking and *n* = 2 for complete pore blocking). The *n* value in Equation (1) can thus be determined from the slope based on a linear correlation between the log(*TMP_t_/dt*) and log(*TMP_t_*). More specific models can be deduced for each fouling mechanism when operating at a constant flux crossflow filtration. In this case, as proposed by Kirschner et al. [25], each specific model can be expressed as follows:

(2) $$\mathrm{Cake}\;\mathrm{layer}\;\Delta TMP=TMP_{0}\left(1+K_{C}Jt\right)$$

(3) $$\mathrm{Intermediate}\;\mathrm{blocking}\;\Delta TMP=\frac{TMP_{0}}{\frac{1}{K_{I}}+\left(1-\frac{1}{K_{I}}\right)\exp\left(-K_{I}Bt\right)}$$

(4) $$\mathrm{Standard}\;\mathrm{blocking}\;\Delta TMP=\frac{TMP_{0}}{{\left(1-K_{S}a_{0}Jt\right)}^{2}}$$

(5) $$\mathrm{Complete}\;\mathrm{blocking}\;\Delta TMP=\frac{TMP_{0}}{1-\frac{\alpha J}{B}\left(1-\;\exp\left(-Bt\right)\right)}$$

where *TMP*_0_ is the initial pressure drop when performing the filtration associated with the intrinsic membrane resistance, *J* represents the flux, *a*_0_ is the unobstructed membrane surface, *α* is the cake specific resistance, *B* is the particle resuspension rate and *K* represents a filtration constant for cake layer (*K_C_*), intermediate blocking (*K_I_*) and standard blocking (*K_S_*). The least squares method was used to adjust the described specific models (Equations (2)–(5)) to the experimental TMP evolution, thus deducing the theoretical parameters from the best fit, while the square of the Pearson correlation (R^2^) and the root-mean-square error (RMSE) were used to identify the model that bests fits the experimental data.

The original permeability of each (virgin) membrane was determined before conducting the experiments. Filtration with tap water was conducted, determining the increase in TMP when raising the operating permeate flux from 5 to 25 LMH, with an increasing step of 4 LMH. Membrane permeability was calculated from Equation (6), estimating it as the average value. Figure S1 (Supplementary Materials) shows the curve obtained for each membrane.

(6) $$K_{exp}=\frac{J}{TMP}$$

NaOCl (2000 ppm) and citric acid (2000 ppm) were used for the membrane chemical cleanings. A cleaning protocol was developed to determine the main fouling source and the chemical cleaning effectiveness of each reagent used, which consisted of the following: after the filtration period, the concentrated sludge was removed from the membrane tanks and substituted by tap water. Different filtering fluxes were applied for at least 60 s (from 5 to 25 LMH with an increasing step of 4 LMH) to determine its influence on the TMP and, consequently, the remaining irreversible membrane fouling. After this process, the membrane modules were cleaned with the basic reagent for at least 8 h, again filling the membrane tank with tap water at the end and repeating the increasing flux-TMP study to assess the membrane permeability recovery. Finally, a similar procedure was performed with the acid reagent, comparing the membrane permeability with the original membranes to assess the effectiveness of the membrane chemical cleaning.

## 3. Results and Discussion

### 3.1. Effect of Membrane Pore Size

**Figure 3:**
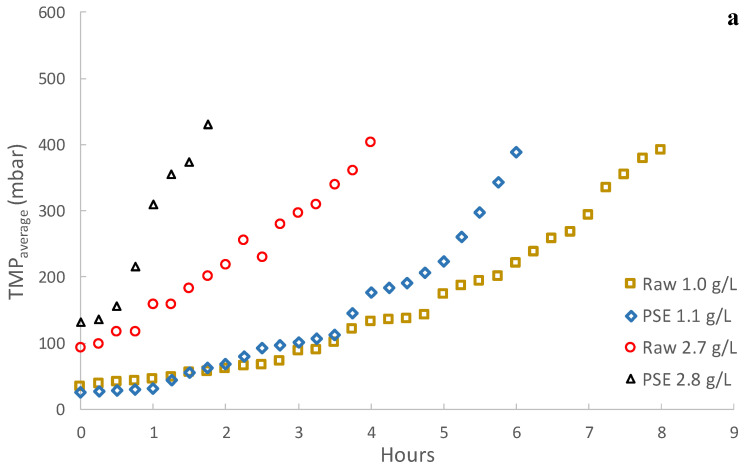

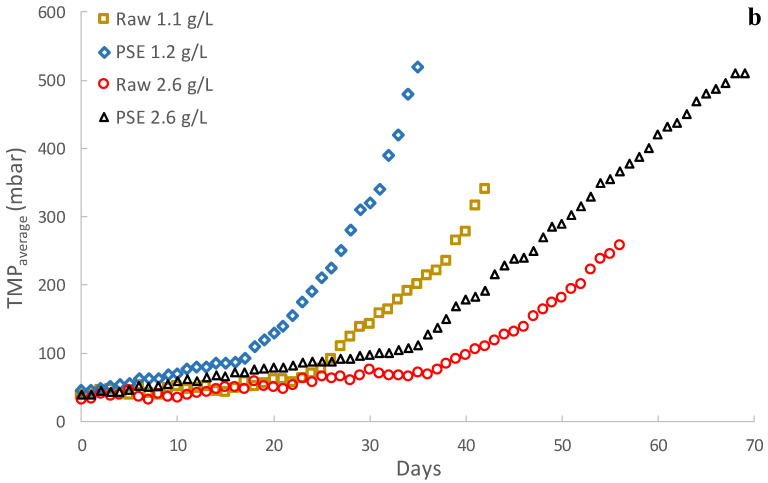
Direct municipal wastewater filtration performance: (**a**) microfiltration membrane and (**b**) ultrafiltration membrane. Note that the legend shows the influent treated together with the average operating total suspended solids concentration. Raw: influent municipal wastewater after a classic pre-treatment (screening and sieving, desanding and degreasing). PSE: effluent of the full-scale wastewater treatment plant primary settler.

Figure 3 shows the results obtained during continuous operation of the plant. In this case study, the MF membrane was only able to operate for a few hours before reaching high TMPs. In this respect, regardless of the different operating conditions tested, severe fouling evolution was observed when using the MF membrane for filtering MWW. A similar performance has been reported in several studies when operating submerged membranes (see, for instance, [18,26,27]), requiring specific fouling control strategies in cited works to dilate filtration (such as coagulant dosing, intensive air sparging or enhanced backwashing). Nonetheless, several studies have demonstrated the potential of MF membranes for MWW treatment when operating in side-stream configuration, where longer filtrations are possible by controlling membrane fouling through cross-flow physical cleaning [28]. As different authors argue, this phenomenon could be due to a thick cake layer formation on the membrane surface [20,21,28] or due to a sticky gel layer on the membrane surface induced by the SMP and EPS content [18,28]. Membrane pore blocking could also occur during the early stage of every filtration cycle before development of the filtration cake layer [20,21]. However, despite the unfavourable results obtained when using the MF membrane, the performance was significantly better when using the UF membrane in similar operating conditions, in this case achieving longer filtering periods thanks to the lower fouling growth rate (see Figure 3b). In fact, two different stages can be appreciated during filtration, a first stable period with no significant irreversible fouling in the first days of operation followed by a second stage in which irreversible fouling consistently increased (see Figure 3b and Table 3). It is important to highlight that the desired TSS concentration was reached in just some hours in all cases so that the concentrating period did not significantly influence the first fouling phase. This dramatic fouling growth difference comparing MF and UF could suggest that the main fouling promotor was related to membrane pore size. Fewer fouling issues could be expected in theory when using higher pore size membranes since less small particulate and colloidal material will be retained on the membrane, reducing the amount of components that could affect the membrane surface or contribute to the cake layer. However, due to the wide particle size range and sticky substances that can be found in untreated MWW, larger pore size membranes would enable more types of material to be deposited in the membrane pores, first promoting pore narrowing, which would evolve until complete blocking. Therefore, use of smaller pore size membrane could be an interesting alternative when filtering untreated MWW since fewer materials would interact with the membrane pores, reducing their blocking propensity.

**Figure 4:**
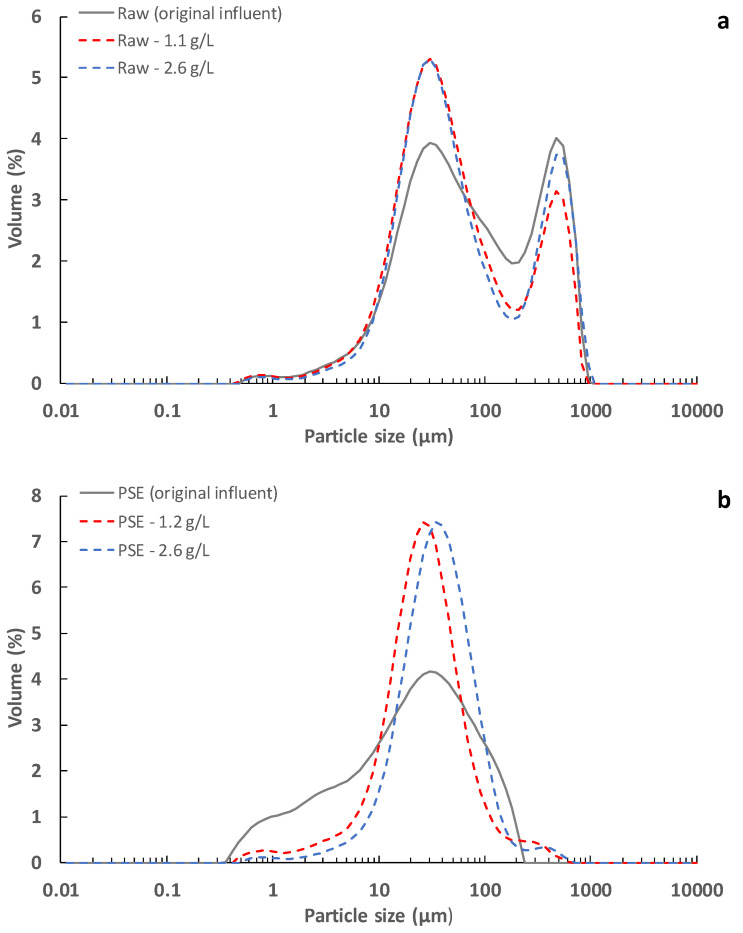
Particle size distribution of the different influents treated during the ultrafiltration membrane operation: (**a**) raw and (**b**) PSE. Note that the legend shows the influent treated together with the average operating total suspended solids concentration. Raw: influent municipal wastewater after a classic pre-treatment (screening and sieving, desanding and degreasing). PSE: effluent of the full-scale wastewater treatment plant primary settler.

To study the influence of the influent pollutants on the membrane, particle size distribution, SMP content, viscosity and filterability of the influents were determined during the UF membrane operation. As can be seen in Figure 4, the particle size distribution of both influents (i.e., raw and PSE) showed that no important particle concentrations formed around the UF membrane (average pore size 0.03 µm), barely observing a small percentage around the MF membrane (average pore size 0.4 µm). According to these results, the particle size of the treated influents should not be able to directly block the pores of the membranes used. On the other hand, despite being soluble compounds, an important retention of the influent SMP content occurred during UF membrane operation (see Table 4). In this regard, Ravazzini [28] reported similar external polymeric substances (EPS) retention capacity when filtering both raw and PSE MWW with an ultrafiltration membrane. These SMP substances could be responsible for a significant fraction of the fouling during filtration, inducing generation of gel layers on the membrane surface, blocking the membrane pores or promoting attachment of other materials to the membrane. Additionally, since both the SMP concentration and filterability of the treated sludge remained constant during each operating period, no important biological effects should be expected. In any case, considering the results obtained from the comparison between the MF and UF membrane technology, use of UF membranes can be strongly recommended for application of the DMF concept. Further studies using smaller pore size membranes (i.e., NF and FO membranes) could thus be suggested for in-depth study of the influence of membrane pore size on fouling growth rate.

### 3.2. Effect of the Influent Used

As Figure 3 shows, lower fouling growth rates were obtained when raw MWW was filtered regardless of the used membrane or the operating TSS concentration. Since only the higher particles (mainly sedimentable solids) were removed from the treated influent when using PSE instead of raw MWW (see Figure 4), these results suggest that, although they are not around the membrane average pore sizes, the smaller size particles are closely related to the fouling mechanisms during filtration. In this case, the treatment of influents with larger particles could contribute to lower fouling growth by (1) reducing the amount of small particles in the mixed liquor and (2) promoting formation of a low-density protective cake layer on the membrane surface. In this regard, when concentrating the particulate fraction of raw MWW, a lower amount of small particles would be interacting with the membrane surface since a higher mass percentage would be covered by the larger particles, thus reducing the pore blocking propensity, especially as the TSS concentration is increased. The presence of larger size particles may also promote formation of more porous cake layers, which could not present a strong resistance to filtration while protecting the membrane surface from direct interaction with colloidal particles or sticky substances. Unfortunately, since similar sludge filterability was obtained regardless of the influent used for each TSS concentration tested (see Table 4), it was impossible to confirm these previous hypotheses. However, since filterability determination uses filters with completely different material and pore size in respect of the employed membranes ones, these results should not be directly compared.

According to the results obtained, use of raw MWW can be recommended for applying the DMF process due to the lower expected irreversible fouling growth rates, at least under the conditions studied in this work. However, further studies considering all the important variables (energy recovery from saved COD, operation and membrane fouling control energy demands, membrane area requirements, chemical cleaning necessities, etc.) need to be performed to properly determine the most favourable conditions for applying this technology considering both the energy and economic point of view as well as environmental aspects.

### 3.3. Effect of Solids Concentration

Different effects were found as the operating TSS concentration was increased depending on the membrane used (see Figure 3). With the UF membrane, a significant improvement was obtained in the filtration performance as the TSS concentration was raised, providing both an extension of the unimportant irreversible fouling evolution period and a reduction in the subsequent irreversible fouling growth rate (see Figure 3 and Table 3). This effect was appreciated regardless of the influent filtered, although the benefits of using raw wastewater seemed to be less marked as the TSS concentration increased. In this case, raising the TSS concentration could promote sporadic flocculation of the small particles by increasing the probability of contact and particle collisions at higher TSS concentrations, thereby reducing the amount of particles able to cause pore blocking or forming a dense cake layer. In this regard, the particle size distribution of the concentrated sludge showed that slightly larger particles can be expected as the TSS concentration is raised for the PSE (see Figure 4b). However, there was a significant reduction in the average particle size when filtering raw wastewater (see Figure 4a). In this respect, it is possible that, due to the compression and shear forces during filtration, a certain aggregate size was promoted. Indeed, a similar predominant particle size was obtained after the filtration process regardless of the original influent. The beneficial effects of increasing the TSS concentration could thus be more related to formation of a protective low-density cake layer on the membrane surface than to the average size of the particles in the treated sludge. Moreover, a slightly increased SMP concentration was found in the treated sludge as TSS concentration was raised (see Table 4). However, this higher SMP concentration did not hinder filtration. According to these results, it can be concluded that either the SMP substances are not as strongly related to the observed fouling as anticipated or that the cake layer formed at higher TSS concentrations is able to protect the membrane from the harmful effects of the SMP compounds [29,30,31]. In fact, the SMP:TSS ratio was importantly reduced as the TSS concentration was raised in the membrane tank (see Table 4), which supports this hypothesis. On the other hand, since using raw MWW showed a similar benefit in fouling control due to the high content of large particles in the treated sludge, increasing the TSS concentration could reduce the influence of the influent fed, reaching a TSS concentration for which the effect of the influent used would be negligible. In this regard, Ravazzini [28] found similar filtration performances regardless of the influent used (raw or PSE wastewater) when operating a UF membrane, concluding that the influent treated is irrelevant. Since increasing the operating TSS showed much better improvement regarding fouling control than the influent used, this strategy can be strongly recommended for direct filtration of MWW. Further studies are required to determine the most favourable TSS concentration considering both the membrane fouling rate and the energy/space requirements for recovery of organic matter. Indeed, since increasing the TSS concentration in the membrane tank would reduce/avoid a later sludge concentration step before injection into the anaerobic reactor, this strategy is recommended for reducing the overall scheme energy and space demands.

A completely different trend was found when increasing the TSS concentration in the MF membrane tank. In this case, a higher irreversible fouling rate was obtained, reducing the filtration time from 8–6 h to just 4–2 h, with a clear benefit when using raw wastewater. As in the UF tank, a slight increase in the average particle size of the filtered mixed liquor could be expected as the TSS rose. However, in this case, the slight increase in the particle size would not be enough to protect the membrane from pore blocking or high-density cake layers, being able even to promote it due to the greater amount of particles with a similar size to the MF membrane pores.

### 3.4. Fouling Control Strategies Effectiveness

**Figure 5:**
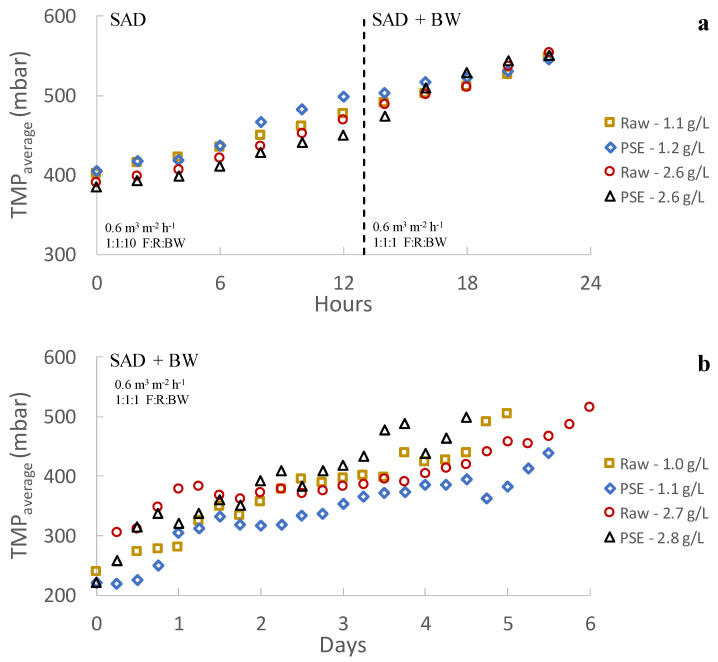
Influence of the applied reversible fouling control strategies (increase in sparging air intensity and backwashing periodicity) on the membrane fouling development: (**a**) ultrafiltration membrane and (**b**) microfiltration membrane. SAD: specific air demand. BW: backwashing. F:R:BW: backwashing periodicity, i.e., filtration:relaxation:backwashing ratio. Raw: influent municipal wastewater after a classic pre-treatment (screening and sieving, desanding and degreasing). PSE: effluent of the full-scale wastewater treatment plant primary settler.

Air sparging and permeate backwashing were used as fouling control strategies. As can be seen in Figure 5, the results obtained when using these strategies depended on the membrane studied. With the UF membrane, neither air sparging intensity nor backwashing frequency were able to significantly reduce the fouling growth rate in any of the operating conditions tested (see Figure 5). These results seem to indicate that the main fouling source when operating the UF membrane was related to irreversible fouling and not to formation of an important reversible cake layer, for which air sparging or backwashing would be effective [32]. It could be assumed, then, that the operating conditions established during continuous filtration (0.1 m^3^ m^−2^ h^−1^ of SAD and 2 min of backwashing every 10 filtration:relaxation cycles) were enough to control the thickness of the developed cake layer as filtration advanced. However, when the same conditions were applied to the MF membrane, there was a significant improvement during filtration, allowing the process to last for at least several days (see Figure 5). This indicated that an important part of the extremely fast membrane fouling developed with the MF module was reversible fouling, which can be minimized by using similar physical cleaning strategies as those used in this study. In fact, other authors have also reported the beneficial effects of using air sparging on filtration performance when filtering MWW with MF membranes [18]. The different influence of the fouling control strategies used on the MF and UF membranes may indicate that different fouling mechanisms were involved. Nonetheless, despite the significant enhancement in MF membrane filtration performance when these strategies were employed, its overall process remained dramatically inferior to the one achieved by the UF membrane. Thus, although physical fouling control strategies would be an interesting option for boosting MF membrane performance when filtering MWW, UF membranes are a more attractive option for applying the DMF of MWW.

### 3.5. Fouling Study

**Figure 6:**
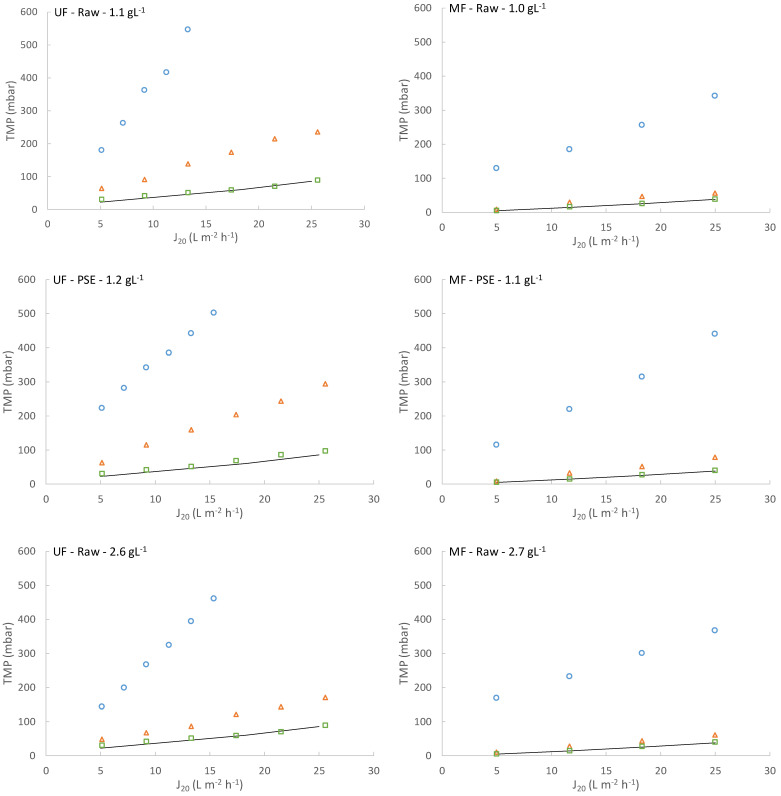

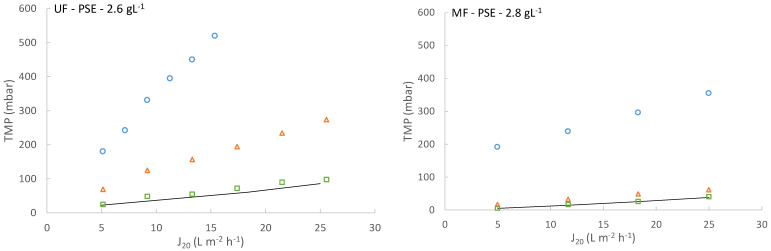
Membrane permeability recovery after the chemical cleaning. Note that the legend shows the membrane used, influent treated and average total suspended solids concentration of each operating period before the chemical cleaning. Tap water was used in all cases for determining the membrane resistance―original membrane resistance; 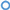 membrane resistance after the operating period; 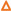 membrane resistance after the basic cleaning; 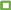 membrane resistance after the acid cleaning. Raw: influent municipal wastewater after a classic pre-treatment (screening and sieving, desanding and degreasing). PSE: effluent of the full-scale wastewater treatment plant primary settler.

Chemical cleaning was performed on both membranes after each set of experiments, studying the permeability recovery of each chemical solution used (i.e., basic and acid solution cleaning). As Figure 6 shows, high fouling removals were obtained after applying the basic solution (2000 ppm of NaOCl), achieving a permeability recovery of about 70–85% and about 92–99% for the UF and MF membranes, respectively. According to these results, organic matter seems to be the main fouling promotor during filtration for both membranes, showing a special relevance for the MF since it was operated during relatively short time periods (around 5–7 days) (see Figure 5). In this regard, other studies have also reported similar results when applying basic solutions for membrane cleaning [17,33,34], also concluding that organic matter was the major fouling promotor during direct MWW filtration. Nonetheless, inorganic fouling (mainly related to inorganic compound precipitation on the membrane) also had a significant effect on UF membrane filtration, which operated for a middle-term (around 1–2 months) (see Figure 3). Although fouling related to organic matter should be the main issue to control during filtration (e.g., by applying chemical-enhanced backwashes with basic solutions), inorganic fouling should also be considered to prolong higher membrane permeability during long-term MWW filtration.

**Figure 7:**
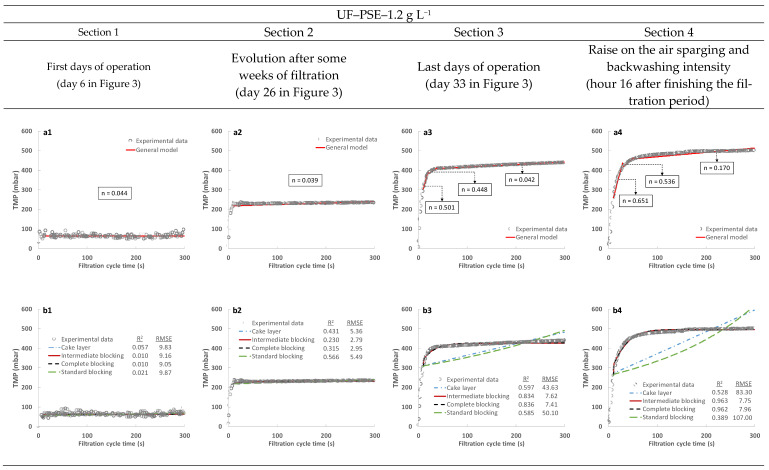

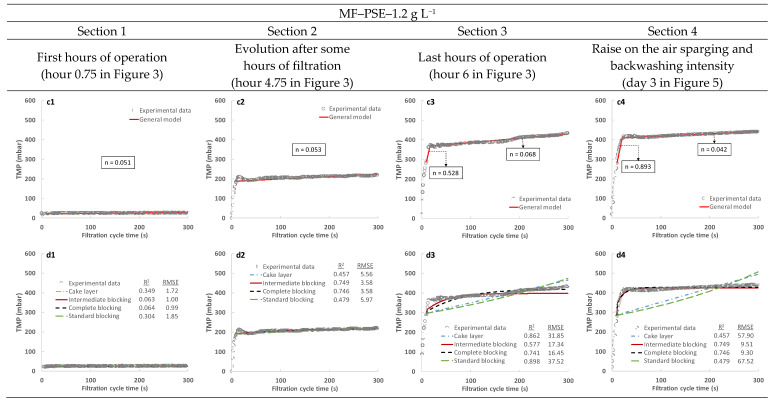
Examples of the fouling mechanism study for the TMP evolution during independent filtration cycles: (**a**,**c**) general model; (**b**,**d**) cake layer, intermediate blocking, complete blocking and standard blocking models. R^2^ and RMSE represent the square of the Pearson correlation and the root-mean-square error, respectively. PSE: effluent of the full-scale wastewater treatment plant primary settler.

All the data concerning TMP evolution during the operation of the plant were adjusted to different theoretical membrane fouling models (Equations (1)–(5)) to study the predominant fouling mechanisms during direct MWW filtration. Since the filtration pump was turned on and the liquid flow rate was adjusted to the set point in the first seconds of every filtration cycle, the first 10 s of data were not considered in the fitting. As can be seen in Figure 7, despite the completely different filtration performances obtained from the MF and UF membranes, there were similar trends when focusing on membrane fouling during each filtration cycle. During the first cycles of operation, both membranes showed low filtration resistance with no significant increase in the TMP as the filtration cycle evolved. Indeed, extremely low R^2^ were obtained when fitting all the mathematical models since an inconsistent evolution of the experimental TMP was found due to some white noise in the sensor signal, although low RMSE values were obtained, indicating a correct fit of the data to all the proposed models. Regarding the general model, close to zero values were obtained, which indicates that cake layer was the predominant fouling mechanism. Since a significant fouling growth was not found during filtration, resistance to filtration would be controlled by the filterability of the treated sludge and the light cake layer formed in the first seconds of each filtration cycle. Regardless of the results obtained during the first filtration cycles, significant irreversible and reversible fouling increases occurred as the operating filtration advanced (see Figure 7, Section 2), which can be divided into two different phases. In the early phase, a significantly higher initial resistance was obtained in respect of the first filtration cycles, although no sludge filterability changes were appreciated (see Table 4). This dramatic increase in filtration resistance was presumably identified as an increment of irreversible fouling due to its quick effect on filtration resistance during the first seconds of every filtration cycle and the inability of air sparging and backwashing to reduce it. This resistance could thus be due to loss of a significant fraction of the useful membrane area caused by pore blocking, gel layer and/or an increment in the compression and robustness of the early formed cake layer as the filtration advanced. After the first seconds of operation, a slight increase in filtration resistance was also found as the filtration cycle advanced. In this second phase, slow reversible fouling was associated with an increment of the formed cake layer thickness on the membrane surface. Indeed, the general model fit produced near to zero values, indicating that the cake layer mechanism was the predominant fouling promotor (see Figure 7(a2,c2)). Additionally, both the cake layer and the standard pore blocking models showed better correlation with the experimental data (see Figure 7(b2,d2)). However, due to the gradual increase in TMP as the filtration cycle advanced, all the models could fit reasonably well with the experimental data, the low correlations obtained being more related to the extremely low slope produced by the intermediate and complete pore blocking models than due to the error between the models and the experimental data, as the low RMSE values obtained confirm. Finally, continuing with the fouling evolution, similar performances were observed as filtration reached high TMPs (see Figure 7, Section 3 and Section 4), accompanied again by two clear fouling phases. In the first phase, there was an important increase in the initial filtration resistance regardless of unchanging sludge filterability, denoting an irreversible fouling increase. In this case, the general model produced *n* values in the range 0.4–0.9 for this phase, indicating that some intermediate blocking could be contributing to fouling (see Figure 7(a3,a4,c3,c4)). However, as commented above, the fouling in this case would be an increment of irreversible fouling during continuous filtration. Indeed, pore blocking generally produces a severe exponential TMP increase as filtration advances without attenuation after reaching a certain point that was obtained during this study. The results produced by the general model could thus be contaminated by this additional accumulated resistance to some degree and should be treated with caution. As in the former case, the second fouling phase seemed completely controlled by cake layer formation as the *n* values were close to zero. However, it is important to highlight that a significant increase in this reversible fouling was obtained as the overall filtration advanced, obtaining higher slopes each time. This phenomenon was probably related to loss of usable membrane area due to irreversible fouling, thereby promoting accumulation of more thick cake layers in non-blocked zones and/or favouring their compression. Concerning the specific models, only the intermediate and complete pore blocking models were able to properly fit the experimental data during these operating sections (see Figure 7(b3,b4,d3,d4)). Nonetheless, as already commented, the evolution of these models is not classic, this trend being forced due to the additional resistance produced by the accumulated irreversible fouling. In fact, the cake layer and standard pore blocking models would fit the experimental data perfectly if the first 30 s of the filtration cycle were not considered (data not shown). Finally, there were no significant differences in model fitting in every operating section when applying this analysis to the data obtained when a different influent (raw or PSE) or TSS concentration (about 1.1 and 2.6 g L^−1^) was employed (data not shown). No relevant differences in the predominant fouling mechanisms during the filtration cycles could thus be expected when operating under the conditions tested in this study.

**Figure 8:**
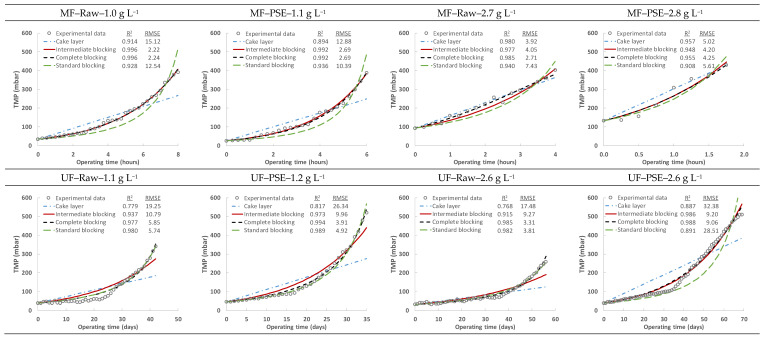
Fitting of the specific fouling mechanism models (cake layer, intermediate blocking, complete blocking and standard blocking models) to the hourly (MF membrane) and daily (UF membrane) TMP evolution obtained during the direct filtration of MWW. R^2^ and RMSE represent the square of the Pearson correlation and the root-mean-square error, respectively. Raw: influent municipal wastewater after a classic pre-treatment (screening and sieving, desanding and degreasing). PSE: effluent of the full-scale wastewater treatment plant primary settler.

Due to the dramatic difference between the MF and UF membrane performance during continuous filtration, despite the similar TMP evolution when only considering independent filtration cycles, all the theoretical models were also applied to the daily (UF membrane) and hourly (MF membrane) TMP evolution exposed in Figure 3 to study/elucubrate the fouling mechanisms involved in the irreversible fouling of these two membranes. As can be seen in Table 5 and Figure 8, the predominant fouling mechanism for both membranes could be considered as intermediate pore blocking, achieving *n* values in the general model around 0.7–1.5 in all cases. The irreversible fouling growth during continuous filtration could thus be due to the same source for both membranes, the different performance between them being exclusively due to the UF membrane capacity to reduce the propensity to fouling. However, the MF membrane data seem to better fit the intermediate and complete pore blocking models, while the UF membrane data, in general, fit better with the complete and standard pore blocking models. These results could indicate that the dramatic difference between the MF and UF membrane performance could be partially due to the different-acting fouling mechanism combination. Intermediate/complete blocking of the pores may occur in the MF membrane during filtration, rapidly hindering its permeability. Instead, UF membrane fouling could be subjected to initial pore narrowing, which could evolve into a complete block as filtration advances, which would explain why fouling of the UF membrane showed two different stages (the first with insignificant irreversible fouling generation related to slight pore narrowing and the second with consistent permeability loss due to complete pore blocking). Nonetheless, it is important to consider that, although one mechanism can govern membrane fouling, they can all occur simultaneously and even change in importance during filtration. Indeed, sharper manifestation of irreversible fouling being observed as filtration advanced was probably related to the state of the membrane after some operational time (i.e., irreversible fouling does not show important effects at the start of filtration since the entire membrane area is available; blocked pores are not so important since a large amount of them are still operative).

From the results obtained from the fouling study, it can be concluded that two different mechanisms control fouling during direct MWW filtration. During the filtration stages, the predominant mechanism would be cake layer formation, which would slightly increase TMP as the filtration cycle advances due to growth in cake layer thickness. This is in agreement with the findings of Fujioka and Nghiem [20] and Ravazzini [28], who also determined that, although pore blocking can occur in the early stage of every filtration cycle, the predominant fouling mechanism is cake layer formation. The low-filtration-resistance cake layer observed in this study may be due to the relatively low operating TSS concentrations tested and/or due to the effectiveness of the employed fouling control strategies (air sparging and backwashing) to control its growth during each filtration cycle. On the other hand, steady accumulation of irreversible organic fouling also occurs as the filtration process continues. In this case, the SMP substances or colloidal organic particles could produce intermediate/complete pore blocking of the membrane pores, in time reducing the useful membrane area dramatically. Fortunately, reducing the membrane pore size or promoting formation of a protecting cake layer on the membrane surface by using raw MWW at higher solids concentrations proved to be effective solutions to reduce this irreversible fouling growth.

## 4. Conclusions

The feasibility of applying the DMF concept for treating MWW was studied on a demonstration plant. The effects of MF and UF membrane technologies, treated influent (raw and PSE MWW) and operating TSS concentration (about 1 and 2.6 g L^−1^) were evaluated. The main findings were as follows:

- Dramatically different performance was obtained depending on the membrane used. Filtration periods of 2–8 h were achieved with the MF membrane, while they were notably increased with the UF module (from 34 to 69 days). This extreme difference was due to the severe fouling when operating the MF membrane, which was dramatically reduced by the UF membrane due to the significantly lower pore size of the latter compared to the former. The benefits observed when operating with a lower membrane pore size were associated with a reduction in pore blocking propensity.
- Both use of raw MWW and increased TSS concentration in the membrane module significantly benefitted the filtration performance with the UF module. This benefit could be associated with the increase in the average particle size, reducing the sludge propensity to block the membrane pores and/or due to formation of a more porous cake layer that acted as a fouling protector.
- The physical fouling control strategies used (air sparging and backwashing) proved to be ineffective in controlling fouling of the UF membrane, although they did have a significant impact on MF membrane fouling, extending the operating time from some hours (2–8 h) to some days (5–6 days).
- The fouling evaluation showed that cake layer formation seemed to be the predominant fouling mechanism during each filtration cycle, representing a reversible fouling increase during filtration. However, as continuous filtration advanced, irreversible fouling appeared. This irreversible fouling could be related to intermediate/complete pore blocking in the case of the MF membrane, while it could also be produced by standard pore blocking in the case of the UF membrane. Organic matter represented more than 70% of this irreversible fouling in all the conditions evaluated.

## Figures and Tables

**Table 1:** Influent municipal wastewater characteristics.

Treated Sewage		Raw	PSE
Parameter	Units	Mean ± SD	Mean ± SD
TSS	mg TSS L^−1^	321 ± 98	132 ± 43
COD	mg COD L^−1^	512 ± 118	195 ± 59
SCOD	mg COD L^−1^	63 ± 28	57 ± 21
SMP	mg COD L^−1^	58.4 ± 11.3	60.9 ± 13.1
TN	mg N L^−1^	56.7 ± 10.8	45.5 ± 8.5
TP	mg P L^−1^	6.4 ± 1.6	5.9 ± 1.1
Alk	mg CaCO_3_ L^−1^	342 ± 73	335 ± 67
pH	-	7.4 ± 0.7	7.6 ± 0.5

**Table 2:** Experimental plan.

Exp. Nomenclature *	Membrane	Sewage Treated	Waste Concentration (g L^−1^)
MF-Raw-1.0	MF	Raw	0.98 ± 0.39
MF-PSE-1.1	MF	PSE	1.09 ± 0.47
MF-Raw-2.7	MF	Raw	2.72 ± 0.68
MF-PSE-2.8	MF	PSE	2.83 ± 0.70
UF-Raw-1.1	UF	Raw	1.11 ± 0.51
UF-PSE-1.2	UF	PSE	1.23 ± 0.43
UF-Raw-2.6	UF	Raw	2.56 ± 0.42
UF-PSE-2.6	UF	PSE	2.63 ± 0.48

* Nomenclature was build quoting in order of membrane system used, treated influent and operating solids concentration in the module.

**Table 3:** Irreversible fouling growth rate during the ultrafiltration membrane operation.

Sewage Treated	Waste Concentration (g L^−1^)	Stable Period (Days)	1st Slope * (mbar/Day)	2nd Slope * (mbar/Day)
Raw	1.1	22	1.81	13.22
PSE	1.2	16	2.87	21.58
Raw	2.6	36	1.13	9.31
PSE	2.6	34	1.99	12.01

* Filtration slopes calculated from a linear regression of experimental data.

**Table 4:** SMP concentration, viscosity and filterability of the treated sludge during the ultrafiltration membrane operation.

Sewage Treated	Waste Concentration (g L^−1^)	SMP Concentration (mg COD L^−1^)	SMP Retention (%)	SMP:TSS Ratio	Viscosity (cSt)	TTF (s)
Raw	1.11 ± 0.51	96.0 ± 14.1	10.6	86.5	0.83 ± 0.04	103 ± 57
PSE	1.23 ± 0.43	78.3 ± 17.8	11.5	65.0	0.82 ± 0.03	79 ± 46
Raw	2.56 ± 0.42	109.1 ± 20.1	16.3	42.6	0.89 ± 0.08	566 ± 178
PSE	2.63 ± 0.48	102.8 ± 22.7	13.8	39.1	0.93 ± 0.08	612 ± 237

**Table 5:** Results of the general fouling model (Equation (1)) for the hourly (MF membrane) and daily (UF membrane) TMP evolution.

Exp. Nomenclature	*n* Value
MF-Raw-1.0	0.978
MF-PSE-1.1	1.194
MF-Raw-2.7	0.893
MF-PSE-2.8	1.515
UF-Raw-1.1	0.717
UF-PSE-1.2	1.405
UF-Raw-2.6	0.689
UF-PSE-2.6	0.761

## Data Availability

Not applicable.

## Supplementary Materials

The following supporting information can be downloaded at: https://www.mdpi.com/article/10.3390/membranes13010099/s1, Figure S1. Original permeability of virgin membranes: (a) MF membrane and (b) UF membrane. Dots represent experimentally determined permeability while the lines the linear fits. TMP: transmembrane pressure, J: Permeate flux, R^2^: square of Pearson correlation, Kexp: membrane permeability average value.
