# Supplementary material for: Direct Membrane Filtration of Municipal Wastewater: Studying the Most Suitable Conditions for Minimizing Fouling Rate in Commercial Porous Membranes at Demonstration Scale
Source: Membranes (Basel). 2023 Jan 12;13(1):99. doi: 10.3390/membranes13010099 (PMC9866899; doi:10.3390/membranes13010099)
Supplement: Supplementary file 1 [file membranes-13-00099-s001.zip › membranes-2116407-supplementary.pdf]

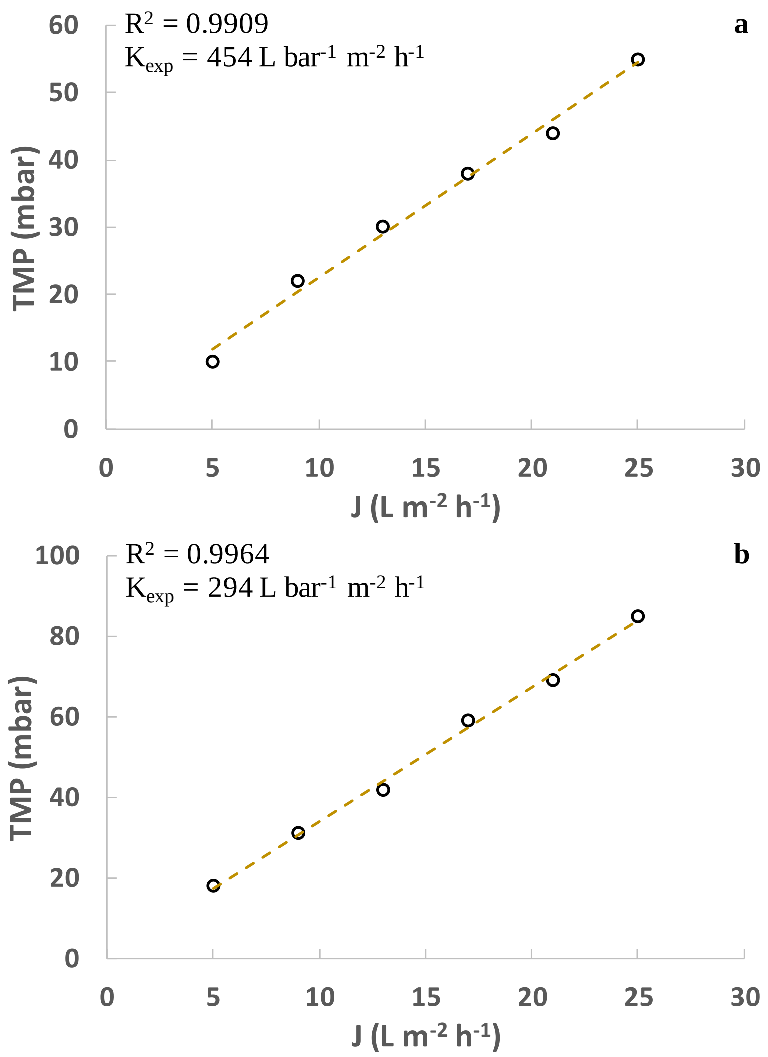

Figure S1. Original permeability of virgin membranes: (a) MF membrane and (b) UF membrane. Dots represent experimentally determined permeability while the lines the linear fits. TMP: transmembrane pressure, J: Permeate flux, R^2: square of Pearson correlation, K_{exp}: membrane permeability average value.
